# Supplementary material for: The solvation shell probed by resonant intermolecular Coulombic decay
Source: Nat Commun. 2024 Aug 13;15:6926. doi: 10.1038/s41467-024-51417-3 (PMC11322543; doi:10.1038/s41467-024-51417-3)
Supplement: Supplementary file 1 — Supplementary Information [file 41467_2024_51417_MOESM1_ESM.pdf]

Supplementary Information: The solvation shell probed by resonant intermolecular Coulombic decay

Rémi Dupuy, Tillmann Buttersack, Florian Trinter, Clemens Richter, Shirin Gholami, Olle Björneholm, Uwe Hergenhahn, Bernd Winter, and Hendrik Bluhm

Supplementary Note 1: Uncertainty in determining peak parameters and sensitivity to the subtraction method

In the main text, in Table 1 we report peak positions and FWHM obtained from fitting the spectra shown in Fig. 4. Here, we describe the uncertainties affecting these numbers. We also address the specific case of the "participator ICD" spectrum (bottom panel of Fig. 4 / last line of Table 1), which is obtained from the subtraction of on- and off-resonance spectra.

Pure-water and off-resonance spectra

Fitting the pure-water and off-resonance spectra (or blue and black traces of Fig. 4 in the main text) gives peak positions and FWHM for the different valence orbitals of water (2nd and 3rd rows of Table 1). The uncertainty of these determinations caused by the fitting procedure is typically small, less than 0.01 eV for the peak positions and 0.02 to 0.05 eV for the FWHM. The $3a_1$ peaks are the least constrained because they are also the least clearly separated and because the fitting forces the same FWHM for both peaks.

The absolute binding energy derived from these peak positions is subject to other sources of uncertainty. The formula for the binding energy is as follows:

$$BE = h\nu + \Delta E_{cutoff} - KE, \tag{1}$$

where KE is the kinetic energy determined from the fit, ΔE_{cutoff} is the position of the low-energy electron cutoff (see the method in detail in Ref. [1]), and $h\nu$ is the photon energy. The accuracy of the cutoff determination is on the order of 0.01 eV [1]. The photon energy dominates the uncertainty here: the beamline monochromator is calibrated using known absorption lines of gases, which, however, are several tens of eV away from our measurement point (∼350 eV). Therefore, we cannot claim a monochromator precision greater than 0.05 eV, which corresponds to the uncertainty of the absolute values given in Table 1 of the main text.

Participator ICD spectrum

Additional considerations come into play for the participator ICD spectrum, which we claim represents the valence-band spectrum of Ca^{2+} hydration-shell water molecules. This spectrum is obtained from the subtraction of two spectra, on- and off-resonance, and we have found that the values and fitting uncertainties of the peak positions and in particular of the FWHM are (i) larger than in the above cases and (ii) very sensitive to the exact way the subtraction is performed.

We show three examples here. In the first case, the normalization of the on- and off-resonance spectra at the Cl^- peak was carried out "visually" by y scaling, y shifting, and x shifting one spectrum to the other, where in the best case the two Cl^- peaks overlap. In the second example, we fitted the on- and off-resonance spectra and obtained peak areas for the Cl^- peak, and normalized both spectra to these peak areas, which is in principle less arbitrary than the first method. This second case is divided into two further cases: we found that the overlap of the Cl^- peaks is not perfect when the spectra are shifted in x by exactly 3.4 eV (the nominal photon-energy difference). The high-KE edge fits better to a shift of 3.38 eV. This deviation of 20 meV is attributed to the non-perfect precision of the monochromator. Nevertheless, we performed a fit for both the 3.38 eV and 3.4 eV shifts. Note that we also found an optimal shift of 3.38 eV for the first method.

The subtracted spectra are shown in Fig. 1 and the fit results are compiled in Table 1. For the $1b_1$ peak, the position does not change significantly and is well constrained. The FWHM, on the other hand, is significantly

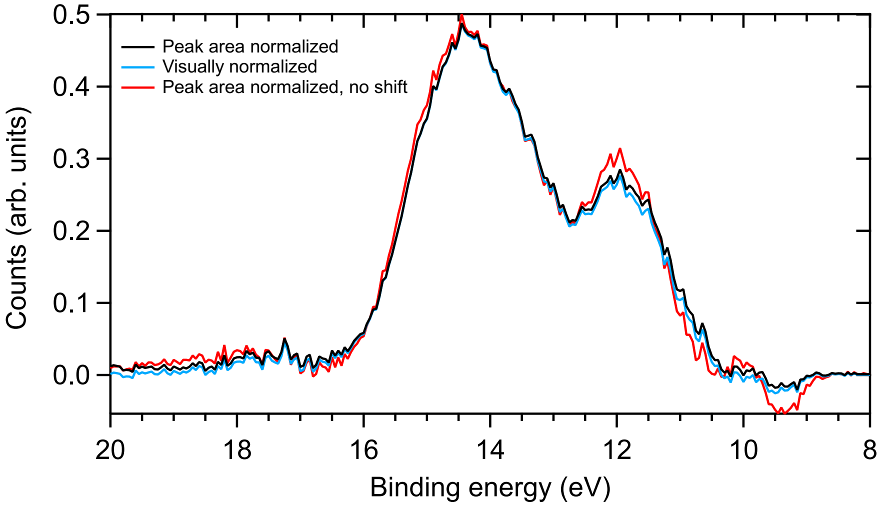

Supplementary Figure 1: Participator ICD spectra obtained using the three different normalization methods detailed in the text.

Supplementary Table 1: Fit results for the three spectra shown in Fig. 1. The uncertainties stated are the fit uncertainties only.

	Peak position (eV)			FWHM (eV)		
	$1b_1$	$3a_1$L	$3a_1$H	$1b_1$	$3a_1$L	$3a_1$H
Peak area normalized	11.85 ± 0.03	13.57 ± 0.23	14.68 ± 0.15	1.47 ± 0.05	1.60 ± 0.21	1.60 ± 0.11
Peak area normalized, no shift	11.88 ± 0.03	13.61 ± 0.22	14.74 ± 0.16	1.42 ± 0.05	1.54 ± 0.3	1.54 ± 0.14
”Visually” normalized	11.84 ± 0.03	13.59 ± 0.16	14.69 ± 0.12	1.59 ± 0.05	1.52 ± 0.21	1.52 ± 0.11

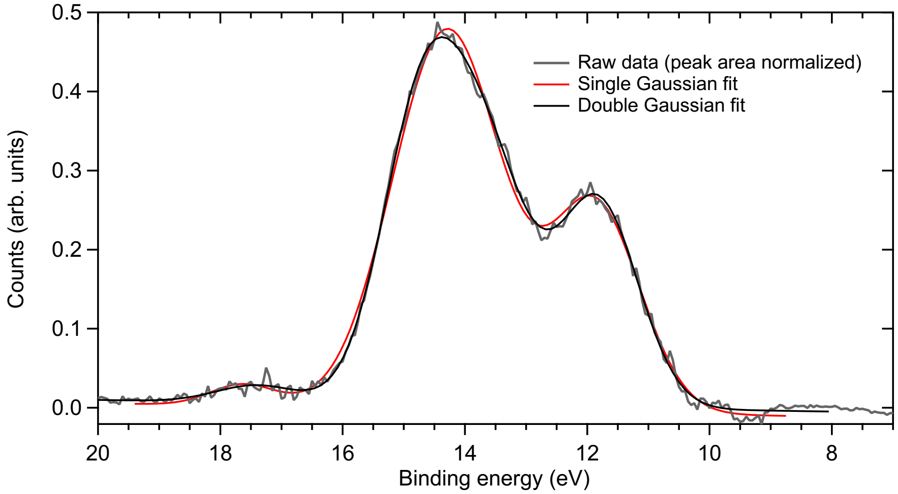

Supplementary Figure 2: Alternative fitting of the participator ICD spectrum (”peak area normalized” from Fig. 1) using a single Gaussian function for the $3a_1$ peak. Fitting with two Gaussian functions is also shown for reference.

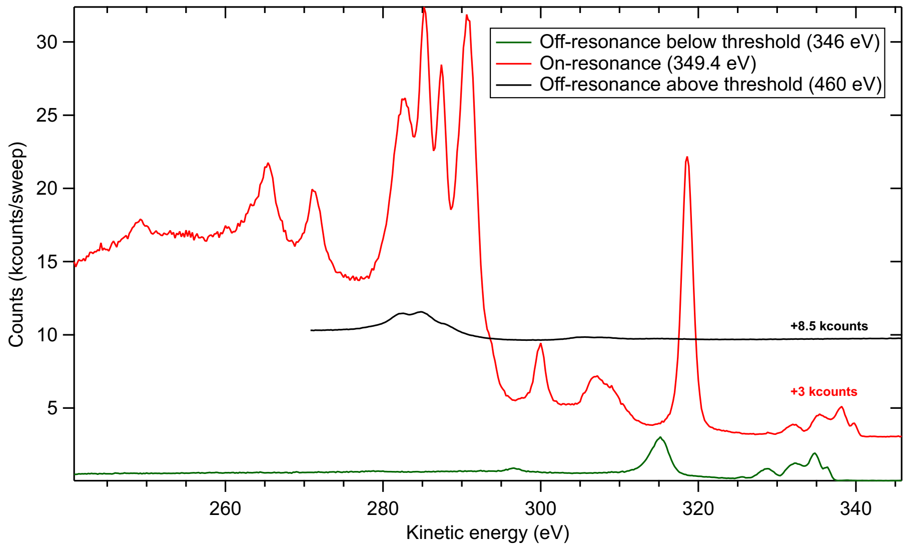

Supplementary Figure 3: Unscaled off- and on-resonance Auger / ICD spectra. The spectra at 349.4 eV and 460 eV photon energies correspond to the (scaled) spectra shown in Fig. 2b of the main text. The y axis shows the raw count number per sweep, where one sweep corresponds to one scan of the detector over the kinetic-energy range and lasts a few minutes. The 460 eV spectrum was recorded with a photon flux about ten times higher than the other two spectra.

influenced by the procedure. One can observe in Fig. 1 that the normalization procedure affects the leading edge of the $1b_1$ peak, i.e., where the peak overlaps with the Cl 3p peak. For this reason, the FWHM reacts very sensitively in this regard. The $3a_1$ peak positions also do not vary much, although the uncertainty derived from the fit is quite large. The same applies to the FWHMs. These results mainly reflect the uncertainty that exists in separating two components of equal width from the broad asymmetric $3a_1$ feature. We attempted to fit this feature with a single, broader Gaussian function. The result is shown in Fig. 2 (red curve, the black curve corresponds to the two-Gaussian fit). Even if the fit is not perfectly adequate, the question is justified as to whether two different $3a_1$ components should be taken into account. Such a question requires theoretical investigations into the nature of the $3a_1$-derived orbital in solvation-shell water molecules.

Supplementary Note 2: Additional spectra

Unscaled resonant and off-resonance Auger / ICD spectra

In Fig. 3, we show the signal enhancement achieved by recording on-resonance spectra. The black and red traces (recorded at 460 eV and 349.4 eV photon energies, respectively) are the same spectra as in Fig. 2b of the main text. The green trace is recorded slightly below the threshold at 346 eV - only the outer- and inner-valence photoelectron lines of water, Cl^-, and Ca^{2+} are visible. Here, the spectra are displayed unscaled as raw count numbers per sweep. In addition to the raw count difference, one should consider the fact that the off-resonance spectrum was recorded with a photon flux about ten times higher than that of the on-resonance spectra. We intentionally reduce the photon flux for high-count measurements, such as resonant measurements, to avoid damaging the electron detector. Overall, the difference between on-resonance and off-resonance at 460 eV is a factor of the order of 200, which makes it much easier to carry out on-resonance measurements.

Evidence for ion pairing in off-resonance ICD spectra

The information about the presence of ion pairing is, in principle, available in all ICD variants (normal, spectator, and participator). In the main text, we argue for the superiority of spectator ICD for detecting ion pairing. We discussed in the main text that the participator ICD is weaker than the spectator ICD and that it has the disadvantage of always appearing on top of the valence photoelectron bands, which requires a normalization and subtraction procedure, which, as described above, can raise problems.

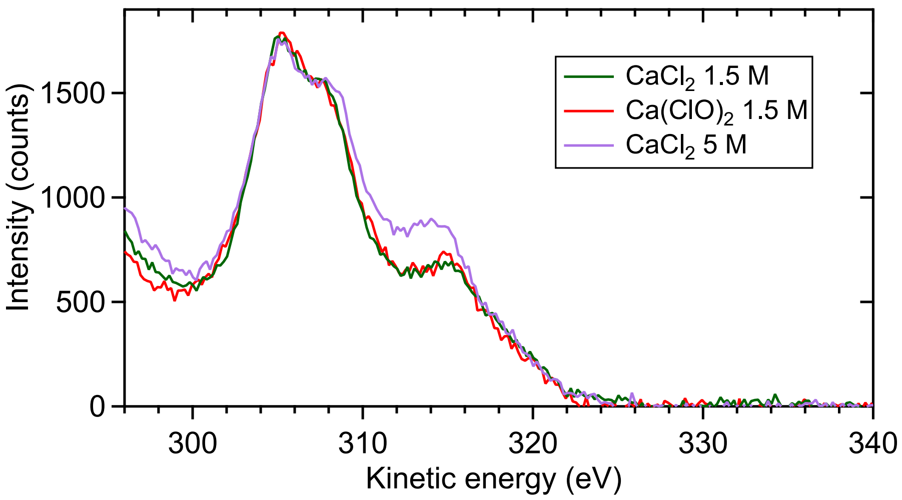

Supplementary Figure 4: Normal ICD spectra at 460 eV for three different solutions.

Here, we show that the ion-pairing information can indeed be observed in off-resonance ICD spectra. In Fig. 4, we show off-resonance ICD spectra recorded at 460 eV photon energy for the 1.5 M and 5 M $CaCl_2$ solutions and for the 1.5 M $Ca(ClO)_2$ solution. At 312 eV kinetic energy, a clear additional signal is observed for the 5 M $CaCl_2$ solution, which we attribute to ICD with Cl^-. This allows the conclusion that ion pairing is present in this solution and not in the other two. The reasoning and conclusions are similar to those developed in the main text for interpreting the spectator ICD spectra.

There are several factors that facilitate ion-pairing detection with resonant spectator ICD. The first is specific to the Ca^{2+} system: one can see that for the 1.5 M $CaCl_2$ solution there is already a large feature peaking around 312 eV and extending down to 320 eV. This feature corresponds neither to ICD with water nor with the counter-ion: it comes from a satellite state (and is therefore not present on-resonance below threshold). We plan to discuss the nature of this state in a future publication. The overlap of this feature with the counter-ion ICD feature makes the latter more difficult to measure.

The other, more general, main factor is that measuring on-resonance significantly enhances the signal, as explained above. The first obvious consequence is the reduction in measurement time: while the spectra shown in Fig. 4 could have been obtained at the cost of several hours of accumulation at the P04 beamline, it would have been difficult to measure them within a reasonable period of time at other less flux-intensive beamlines, in contrast to the on-resonance spectra. The second, less obvious consequence is that even at high flux, weak signals are difficult to detect because they lie on a large background and can be obscured by overlap with photoelectron lines that happen to appear at the same energies. It is possible to some extent to tune out these photoelectron lines by changing the photon energy, but their influence extends over a large range, since even the inelastic-scattering tail associated with each line, which extends at least 20 eV below the line, can obscure a weak signal. Precisely for this reason, we could not properly measure the normal ICD spectra at 460 eV photon energy of the CaI_2 and $Ca(NO_3)_2$ solutions, due to interference of, respectively, the I 4p/4s lines and the N 1s Auger features.

Resonant spectator ICD at lower $CaCl_2$ concentrations

In Fig. 5, we plot additional resonant spectator ICD spectra from 0.7 M and 1.4 M $CaCl_2$ solutions. The two curves show similar spectator ICD characteristics within the signal-to-noise ratio of the measurement. This experiment shows that there is no contribution from Cl^- to the spectator ICD signal in the 1.5 M spectrum reported in the main text - otherwise a progressive growth of the feature observed at 312 eV kinetic energy at high concentration would already be visible from 0.7 M to 1.4 M. The spectra of Fig. 5 were recorded at the PLEIADES beamline of the SOLEIL synchrotron with a different liquid-microjet setup than that at P04. Since the light polarization is different in both cases (circular at P04 and horizontal at PLEIADES), we did not attempt to make further comparisons between these spectra and those recorded at P04.

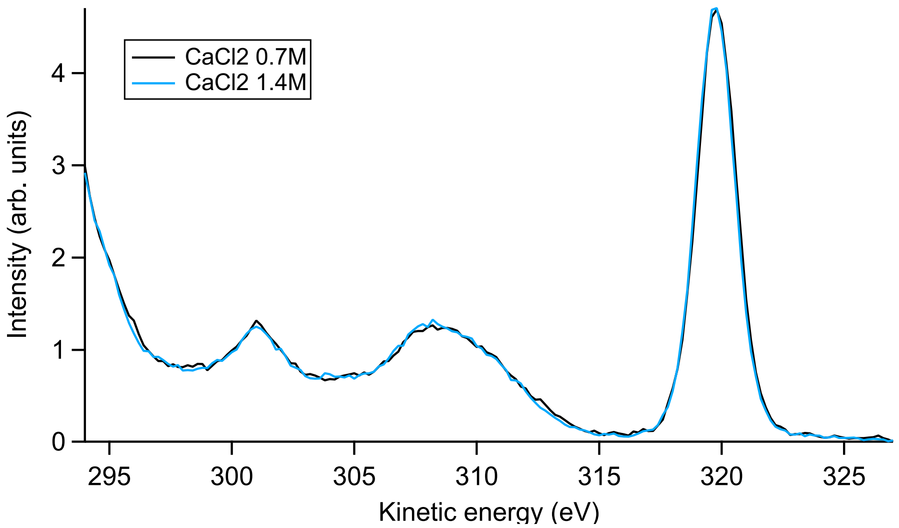

Supplementary Figure 5: Additional spectra to Fig. 3 of the main text, showing spectator ICD for 0.7 M and 1.4 M $CaCl_2$ solutions.

References

[1] S. Thürmer, S. Malerz, F. Trinter, U. Hergenhahn, C. Lee, D. M. Neumark, G. Meijer, B. Winter & I. Wilkinson; "Accurate vertical ionization energy and work function determinations of liquid water and aqueous solutions"; Chemical Science **12**, pp. 10558–10582 (2021).
